# Improving the Junior Doctor Induction Programme in an NHS Trust

**DOI:** 10.1192/bjo.2025.10420

**Published:** 2025-06-20

**Authors:** Udoka Onyechere, Harvir Sahota, Seetal Chavda, Toibat Adeyinka

**Affiliations:** 1 Cumbria, Northumberland, Tyne and Wear Foundation NHS Trust, Newcastle, United Kingdom; 2 Coventry and Warwickshire Partnership NHS Trust, Coventry, United Kingdom

## Abstract

**Aims:** The aim of this Quality Improvement (QI) project was to enhance the induction programme for junior doctors at an NHS Trust, ensuring it is more meaningful and better prepares trainees for their psychiatry rotation.

**Methods:** Surveys were conducted with junior doctors during their rotations to identify areas of dissatisfaction and potential improvements within the induction process. The feedback was subsequently analysed to develop and implement targeted interventions. These interventions included the modification of a local induction programme schedule led by junior doctor representatives and other key leaders within the Trust, revision of the Trust junior doctor handbook to incorporate up-to-date practical and rotation-specific guidance, and creation of an induction pack hosted on Microsoft Teams available to all junior doctors joining the Trust. Key quality improvement tools, including driver diagrams, questionnaires, measures checklist and Plan-Do-Study-Act (PDSA) cycles, were employed to evaluate and refine the implemented changes.

**Results:** Survey responses demonstrated significant improvements in preparedness for psychiatry rotations and comprehension of roles and responsibilities post-intervention. Specifically, there was a two-fold increase in the proportion of trainees reporting preparedness for their rotation, from 33% pre-change to 66% post-change. Similarly, those who reported understanding their roles and responsibilities increased from 35% to 65%. Notwithstanding these improvements, persistent challenges include the inability to fundamentally alter the overarching three-day trust-wide induction and difficulties in assessing the sustained impact of changes due to high turnover among trainees.

**Conclusion:** This project addressed key deficiencies in the induction programme for junior doctors in the Trust, demonstrating that targeted, trainee-led changes can significantly improve preparedness for their psychiatry rotation. Future efforts would focus on embedding sustainable improvements and exploring further restructuring of the broader trust-wide induction programme to address systemic issues.

